# INSTRUMENTS MEASURING SELF-CARE BEHAVIORS OF OLDER ADULTS WITH CHRONIC KIDNEY DISEASE: A SCOPING REVIEW

**DOI:** 10.1093/geroni/igae098.0495

**Published:** 2024-12-31

**Authors:** Thanakrit Jeamjitvibool, Wirampa Tanglai

**Affiliations:** University of Illinois Chicago, Chicago, Illinois, United States; University of Illinois Chicago, Chicago, Illinois, United States

## Abstract

Promoting self-care behavior among older adults with chronic kidney disease (CKD) is critical for preventing kidney deterioration, particularly in the early stages, as symptoms often become noticeable only in later stages. Numerous assessment tools exist, but their suitability and quality remain uncertain. This review aims to identify, assess methodologies, and consolidate psychometric properties of self-care assessment instruments for older adults with CKD. We followed Askey & O’Malley’s (2005) scoping methodology and PRISMA-ScR guidelines. PubMed, Scopus, CINAHL, PsycInfo, and Embase were searched until October 5, 2023. Included studies tested measurement properties of self-reported instruments assessing self-care behaviors in older adults with CKD, were published in English, and appeared in peer-reviewed journals. The COSMIN framework was used to appraise study quality. Ten articles addressing instruments for self-care behaviors in older adults with CKD were included. Based on COSMIN, no article reported complete measurement properties. Six instruments were developed with theory support, while no theoretical underpinning was identified for three instruments. Only one instrument was developed based on qualitative research. Some instruments were applied to a combined population, such as patients with diabetes and CKD. Although there have been advances in creating tools for assessing self-care behaviors in older adults with CKD, no single instrument fully reported measurement properties. Decision-making regarding which of the available tools is appropriate for practice or research should consider their intended purpose, scope, and any underlying theoretical principles. Future research should focus on developing a comprehensive instrument for CKD self-care behaviors that exhibits high methodological quality.

